# Comparative Anterior Pituitary miRNA and mRNA Expression Profiles of Bama Minipigs and Landrace Pigs Reveal Potential Molecular Network Involved in Animal Postnatal Growth

**DOI:** 10.1371/journal.pone.0131987

**Published:** 2015-07-02

**Authors:** Rui-Song Ye, Meng Li, Qi-En Qi, Xiao Cheng, Ting Chen, Chao-Yun Li, Song-Bo Wang, Gang Shu, Li-Na Wang, Xiao-Tong Zhu, Qing-Yan Jiang, Qian-Yun Xi, Yong-Liang Zhang

**Affiliations:** Chinese National Engineering Research Center for Breeding Swine Industry, SCAU-Alltech Research Joint Alliance, Guandong Provincial Key Lab of Agro-Animal Genomics And Molecular Breeding, College of Animal Science, South China Agricultural University, Guangzhou, 510642, China; Universidade de Sao Paulo, BRAZIL

## Abstract

The anterior pituitary is the most important endocrine organ modulating animal postnatal growth, mainly by controlling growth hormone (GH) gene transcription, synthesis, and secretion. As an ideal model for animal postnatal growth studies, the Bama minipig is characterized as having a lower growth performance and fewer individual differences compared with larger pig breeds. In this study, anterior pituitaries from Bama minipig and Landrace pig were used for miRNA and mRNA expression profile analysis using miRNA microarrays and mRNA-seq. Consequently, a total of 222 miRNAs and 12,909 transcripts were detected, and both miRNAs and mRNAs in the two breeds showed high correlation (r > 0.97). Additionally, 41 differentially expressed miRNAs and 2,254 transcripts were identified. Pathways analysis indicated that 32 pathways significantly differed in the two breeds. Importantly, two GH-regulation-signalling pathways, cAMP and inositol 1, 4, 5-triphosphate (IP3), and multiple GH-secretion-related transcripts were significantly down-regulated in Bama minipigs. Moreover, TargetScan and RNAHybrid algorithms were used for predicting differentially expressed miRNAs (DE miRNAs) and differentially expressed mRNAs (DE mRNAs) interaction. By examining their fold-changes, interestingly, most DE miRNA–DE mRNA target pairs (63.68–71.33%) presented negatively correlated expression pattern. A possible network among miRNAs, mRNAs, and GH-regulation pathways was also proposed. Among them, two miRNA-mRNA interactions (Y-47 targets FSHB; ssc-miR-133a-3p targets GNAI3) were validated by dual-luciferase assay. These data will be helpful in understanding the possible molecular mechanisms involved in animal postnatal growth.

## Introduction

The pituitary (to be more exact, the anterior pituitary) is the most important endocrine organ regulating animal postnatal growth due to its central role in the growth axis[1]. It is well known that the pituitary exerts growth-promoting actions that are dependent on growth hormone (GH): pituitary somatotropes integrate complicated extracellular signals from the hypothalamus (such as GHRH, SS)[2], the feedback of target organs (such as the liver)[3], as well as paracrine regulation (such as luteinizing hormone, LH)[4], and then lead to intracellular signalling pathways modulating GH gene transcription, hormone synthesis, and secretion. Obviously, GH synthesis and secretion regulation within the pituitary are determined by thousands of molecules from multiple levels, including mRNAs at the transcriptional level and miRNAs at the post-transcriptional level.

Transcriptional regulation of GH within the pituitary has been extensively studied over decades. Previous studies revealed a variety of genes and pathways involved in GH gene regulation, such as *GH1* genes, transcription factor *POU1F1* (*Pit-1*), *SP-1*, and *CREB*[5, 6], as well as signalling pathways, cAMP signalling (adenylate cyclase/cAMP/protein kinase A), and IP3 signalling (phospholipase C-IP3-protein kinase C signal pathways)[7, 8]. MicroRNAs (miRNAs) are small (~21-nt) but powerful non-coding RNAs that post-transcriptionally regulate gene expression, mainly by binding to the 3′-untranslated region (3′-UTR) of its target mRNAs, reducing mRNA transcription or translation[9, 10]. Evidence of the importance of pituitary miRNAs involved in GH regulation is increasing; for example, miR-26b regulated GH by targeting *LEF-1*, a repressor of POU1F1[11]. A previous study demonstrated that miR-34b, miR-326, miR-432, miR-548c-3p, miR-570, and miR-603 may participate in human GH adenomas[12]. Collectively, pituitary GH is regulated by thousands of molecules at both the transcriptional and the post-transcriptional levels.

The pig (*Sus scrofa*) is an important meat source in human food[13] and it is also a good biomedical model due to the similarities in anatomy and physiology with humans, especially the miniature pig[14]. The Bama minipig is a Chinese local miniature pig breed that has been frequently used in biomedical studies[15]. Compared with European pig breeds (such as Landrace pigs), Bama minipigs are characterized as having fewer individual differences (highly inbred) and lower growth performance (average adult weight: 50–60 kg)[16]. Therefore Bama minipigs are ideal models for studying animal growth. A previous study has investigated the pituitary gene-expression profiles of two miniature pig breeds (Bama minipigs and Tibetan minipigs) at different postnatal development stages[17], but little is known of the anterior pituitary mRNA as well as miRNA expression differences between minipigs and large pigs at the whole-genome level. In this present study, we have focused on the anterior pituitary, and analysed miRNA and mRNA expression profiles between Bama minipigs and large pigs (Landrace) using miRNA microarray and mRNA-Seq in order to identify possible molecules (miRNAs and mRNAs) and pathways that are involved in animal postnatal growth regulation. The potential networks among miRNAs, mRNAs, and signalling pathways were also investigated.

## Materials and Methods

### Ethics Statement

The animal slaughter experiments were conducted in accordance with the guidelines of Guangdong Province on the Review of Welfare and Ethics of Laboratory Animals approved by the Guangdong Province Administration Office of Laboratory Animals (GPAOLA). All animal procedures were conducted under the protocol (SCAU-AEC-2010-0416) approved by the Institutional Animal Care and Use Committee (IACUC) of South China Agricultural University.

### Animal

Three 20-day-old healthy female Landrace (YC) and Bama minipig (YB) piglets were used. The average body weight of Landrace cohort was 6.43±0.55 kg; and the average body weight of Bama minipig cohort was 3.24 ± 0.88 kg, so a significant difference in the body weight was observed between YC and YB.

### RNA extraction and quality assessment

Briefly, under sterile conditions, pituitary glands were removed and the anterior lobe was immediately dissected from each pituitary gland. Anterior pituitary glands were washed in PBS solution for about 5–10 s, in order to reduce blood contamination. Three anterior pituitary glands were immediately frozen in liquid nitrogen until RNA extraction. Total RNA was extracted from the anterior pituitary tissue using TRIzol reagent (Invitrogen, Carlsbad, CA, USA) following the manufacturer’s instructions. RNA quality and quantity of all samples were evaluated by using a ND-2000 nanodrop spectrophotometer (NanoDrop Technology,Wilmington,DE). Total RNAs were assessed further for RNA integrity on the Agilent Bioanalyzer (Santa Clara, CA), and all samples had an RNA Integrity Number (RIN) of 7 or better. Qualified RNAs were used for further miRNA microarray analysis and mRNA-seq analysis.

### miRNA probe design and microarray assay

Custom-designed μParaflo microfluidic chips (LC Sciences, Houston, TX) were used for miRNA expression analysis. In this study, we used all porcine miRNAs deposited in miRBase 18.0 and designed sequences as miRNA microarray probes. In detail, miRBase 18.0 collected a total of 257 porcine miRNAs (255 miRNAs according to its sequence). We added 100 probes (probes labelled with prefix ‘Y-’) according to our previous reports[18, 19] and miRNA sequence homology with other species (human, mouse, rat). In addition, precursor sequences of these miRNAs were also analysed (see S1 Table) and the mature miRNA sequences confirmed through sequencing (unpublished data). All probes for the microarray were made by *in situ* synthesis using PGR (photogenerated reagent) chemistry.

Microarray assays were obtained using a service provider (LC Sciences, Houston, TX). In brief, 2–5 μg total RNA samples were used for small RNAs (<300 nt) enrichment by a YM-100 Microcon centrifugal filter (Millipore, Billerica, MA); the collected small RNAs were then 3′-extended with a poly (A) tail using poly (A) polymerase and an oligonucleotide tag was ligated to the poly (A) tail for later fluorescent dye staining. Hybridization was performed overnight on a μParaflo microfluidic chip using a micro-circulation pump (Atactic Technologies, Houston, TX). After hybridization, detection was performed by fluorescence labelling using tag-specific Cy5 dyes. Hybridization images were collected using a laser scanner (GenePix 4000B, Molecular Device, Silicon Valley, CA) and digitized using Array-Pro image analysis software (Media Cybernetics, Bethesda, MD). Data were analysed by first subtracting the background and then normalizing the signals using a LOWESS filter (locally weighted regression). miRNAs had to meet at least three conditions to be considered detectable: a signal intensity higher than 3× (background standard deviation), a spot CV < 0.5 calculated by (standard deviation)/(signal intensity), and a p-value ≤ 0.01. We used Log2|fold-change| ≥ 1, p ≤ 0.01(statistical significance was determined by Student's t test.) as the cut-off for filtering the differentially expressed miRNAs between the two groups. The miRNA microarrays data have been submitted to the GEO database under accession number GSE68489 (GSM1673695, GSM1673696).

### mRNA analysis by mRNA-seq

The anterior pituitary mRNAs (transcripts) of Bama minipigs and Landrace pigs were analysed using the mRNA-seq technique. In brief, qualified total RNAs were prepared for the cDNA library construction; the library products were prepared for sequencing analysis via an Illumina HiSeq2000, in order to obtain raw sequence data. To obtain clean reads, adaptor contamination, those in which unknown bases numbered more than 10, and low-quality reads (where the percentage of low-quality bases of quality value ≤ 5 was more than 50% in a read) were removed. Clean reads were further mapped to reference genome (ftp://ftp.ensembl.org/pub/release-65/fasta/sus_scrofa/dna/Sus_scrofa.Sscrofa9.65.dna.toplevel.fa.gz) and gene databases (ftp://ftp.ensembl.org/pub/release-65/fasta/sus_scrofa/cdna/Sus_scrofa.Sscrofa9.65.cdna.all.fa.gz) using SOAPaligner/soap2; mismatches of no more than two bases were allowed in the alignment. After assessment of sequencing (including sequence read quality, statistics of alignment analysis, sequencing saturation analysis, distribution of reads on reference genes/genome), the gene-expression level was calculated using the RPKM method (Reads Per kb per Million reads)[20]. The significance of differentially expressed genes among samples was determined according to Audic and Claverie[21]. We used false discovery rate (FDR) ≤ 0.001 and the absolute value of log2Ratio(YB/YC) ≥ 1 as the threshold for judging the significance of the gene-expression difference; differentially expressed genes were further employed to KEGG pathway analysis. All mRNA-seq assays and analyses were conducted by the Beijing Genomics Institute (BGI, Shenzhen, China), the mRNA-Seq data have been submitted to the GEO database under accession number GSE68490 (GSM1673697, GSM1673698).

### qRT–PCR validation of miRNAs and mRNAs

To validate the microarray data and mRNA-seq data, 2 μg total RNAs of each sample of Bama minipigs and Landrace pigs were transcribed to cDNA using the One Step PrimeScript miRNA cDNA Synthesis Kit (Takara, Dalian, China) according to methods as described by our previous study[22]. To quantify miRNA and mRNA expressions of each group, cDNA was diluted 5-fold with ddH_2_O; a final 20-μl volume qRT–PCR reaction was performed on a STRATAGENE Mx3005P sequence detection system. The PCR Reaction mix consisted of 2 μl cDNA, 10 μl 2× SYBR Green PCR Master Mix (Toyobo, Osaka, Japan), and 10 uM of each primer. The thermal profile of real-time PCR was as follows: 1 min at 95°C, 40 cycles of 15 s at 94°C and 15 s at the corresponding annealing temperature (Tm), and 72°C for 40 s, followed by a quick denaturation at 95°C for 5 min, Tm, plus a slow ramp from Tm to 95°C to generate a melt curve to control the specificity of the amplified product. NTC (no template control) was set as the negative control for each miRNA and mRNA; all reactions were performed in triplicate. For all the differentially expressed miRNAs, the U6 small nuclear RNA was used as an internal control. The 2-ΔCt method was employed to quantify and normalize the expression data. For the mRNA validation, gene expression was examined using the same template, volume and thermal reaction conditions, using β-actin gene as the control. All primers were designed by Primer 5.0; information about primers is listed in S2 Table.

### Differentially expressed miRNA–mRNA interaction prediction

RNAhybrid algorithm (https://bibiserv2.cebitec.uni-bielefeld.de/rnahybrid) and TargetScan algorithm (www.targetscan.org/) were employed to analyse the potential miRNA–mRNA target relationships. In brief, we obtained all differentially expressed porcine transcripts 3′-UTR from the Ensemble database (www.ensembl.org/). In RNAhybrid algorithm analysis, a cut-off ‘perfect match of 2–8 seed sequence and –25 kcal/mol thermal energy, G: U matches allowed’ was employed. For the TargetScan prediction, the default parameters were used. Due to the negative regulatory role of miRNAs, the proportions of negatively correlated miRNA–mRNA pairs (up-regulated miRNA–down-regulated mRNA or down-regulated miRNA–up-regulated mRNA pairs were considered) in RNAhybrid- and/or TargetScan-predicted miRNA–mRNA target pairs were calculated. We plotted the potential network between miRNAs and mRNAs using Cytoscape3.2.0(http://www.cytoscape.org/)[23].

### Dual-luciferase miRNA target expression vector

Based on the predicted miRNA-mRNAs results, *FSHB* 3′-UTR bearing ssc-miR-7139-3p seed binding site and *GNAI3* 3′-UTR bearing ssc-miR-133a-3p seed binding site (S1 Text) were generated by two complementary chemically synthesized oligos (Sangon,Shanghai,China), respectively. The complementary oligonucleotides were resuspended at a 1:1 ratio (1 μg/μL each) in an annealing buffer (10 mM Tris, pH 7.5–8.0, 50 mM NaCl, and 1 mM EDTA) and heated to 95°C for 10 min to remove secondary structures. The temperature was then gradually reduced until room temperature was reached. The annealed products were then cloned into the pmirGLO vector (Promega) downstream from the firefly luciferase coding region (Xho I and Xba I sites).

### Luciferase reporter assay

CHO cells were seeded in 48-well cell culture plates (4 × 10^4^ cells per well), and cultured in RPMI 1640 (Life Technologies, Grand Island, NY) supplemented with 10% FBS. The next day, the cells were firstly transfected with recombinant pmirGLO-3′-UTR vector (200ng/well) using Lipofectamine 2000 (Life Technologies, Grand Island, NY) as described by the manufacturer for adherent cell lines. Six hours after transfection, the second transfection were performed with their corresponding miRNAs mimics (ssc-miR-7139-3p for FSHB, ssc-miR-133a-3p for GNAI3) or a negative control (10pmol/well, GenePharma, Shanghai, China). Cells were harvested 48h after the second transfection, and luciferase activity was determined using a dual luciferase reporter assay system, according to the manufacturer’s recommendations (Promega, Madison, WI). Normalized firefly luciferase activity (firefly luciferase activity/Renilla luciferase activity) for each construct was compared with that of the pmirGLO vector.

## Results

### miRNA expression in the anterior pituitary of Bama minipigs and Landrace pigs

**Fig 1:**
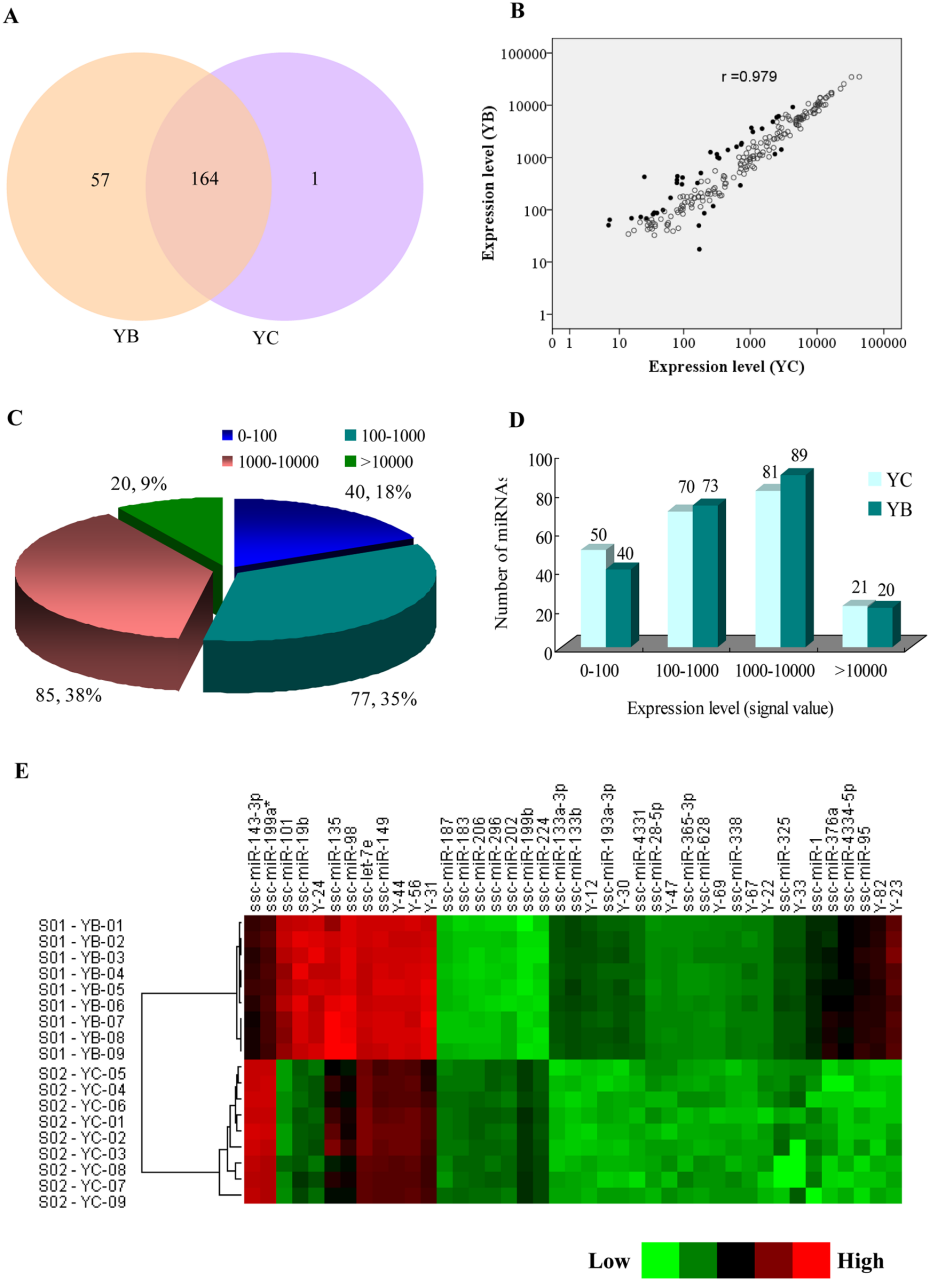
miRNA expression and distribution. (A) miRNA numbers of two pig breeds; YB and YC indicate Bama minipigs and Landrace pigs, respectively. (B) Scatter plots of miRNA expression; *r* represents correlation between Bama minipigs and Landrace pigs and the solid dots represent significantly changed miRNAs. (C) The average expression levels of miRNAs and their corresponding proportion in the anterior pituitary. (D) The miRNA numbers of the two pig breeds at different expression levels. (E) Cluster analysis of differentially expressed miRNAs between YB and YC. YB and YC indicate Bama minipigs and Landrace pigs, respectively.

Initially, we examined the miRNA expression of Bama minipigs (YB) and Landrace pigs (YC) using custom-designed miRNA microarrays; 355 miRNAs were designed and examined by miRNA array (nine replications per miRNA), and 222 miRNAs were detectable (Fig 1A), which include 172 annotated miRNAs and 50 miRNAs designed by us (probes labelled with the prefix ‘Y-’) (S3 Table). Correlation analysis indicated that global miRNA expressions in the two breeds is quite similar (Pearson’s correlation coefficient, *r* = 0.979 in Bama minipigs and Landrace pigs) (Fig 1B). Of these, 40 miRNAs are minimally expressed (0 < average signals ≤ 100; p ≤ 0.01), 77 miRNAs are modestly expressed 100 < average signals ≤ 1,000; p ≤ 0.01), 85 miRNAs were highly expressed (1,000 < average signals ≤ 10,000), and, in particular, 20 miRNAs were extremely highly expressed in the anterior pituitary (average signals ≥ 10,000; p ≤ 0.01), including ssc-miR-7, Y-90, ssc-miR-26a, ssc-miR-125b, Y-1, ssc-miR-125a, Y-77, ssc-let-7g, ssc-miR-29a, ssc-let-7i, ssc-let-7a, ssc-let-7f, ssc-miR-148a, ssc-miR-21, ssc-miR-335, ssc-miR-30b-5p, ssc-miR-191, ssc-miR-29c, ssc-miR-23b, and ssc-miR-23a (Fig 1C). There were no obvious differences in the miRNA numbers between the two breeds in terms of the signal value range (Fig 1D).

### Differentially expressed miRNAs between Bama minipigs and Landrace pigs

**Fig 2:**
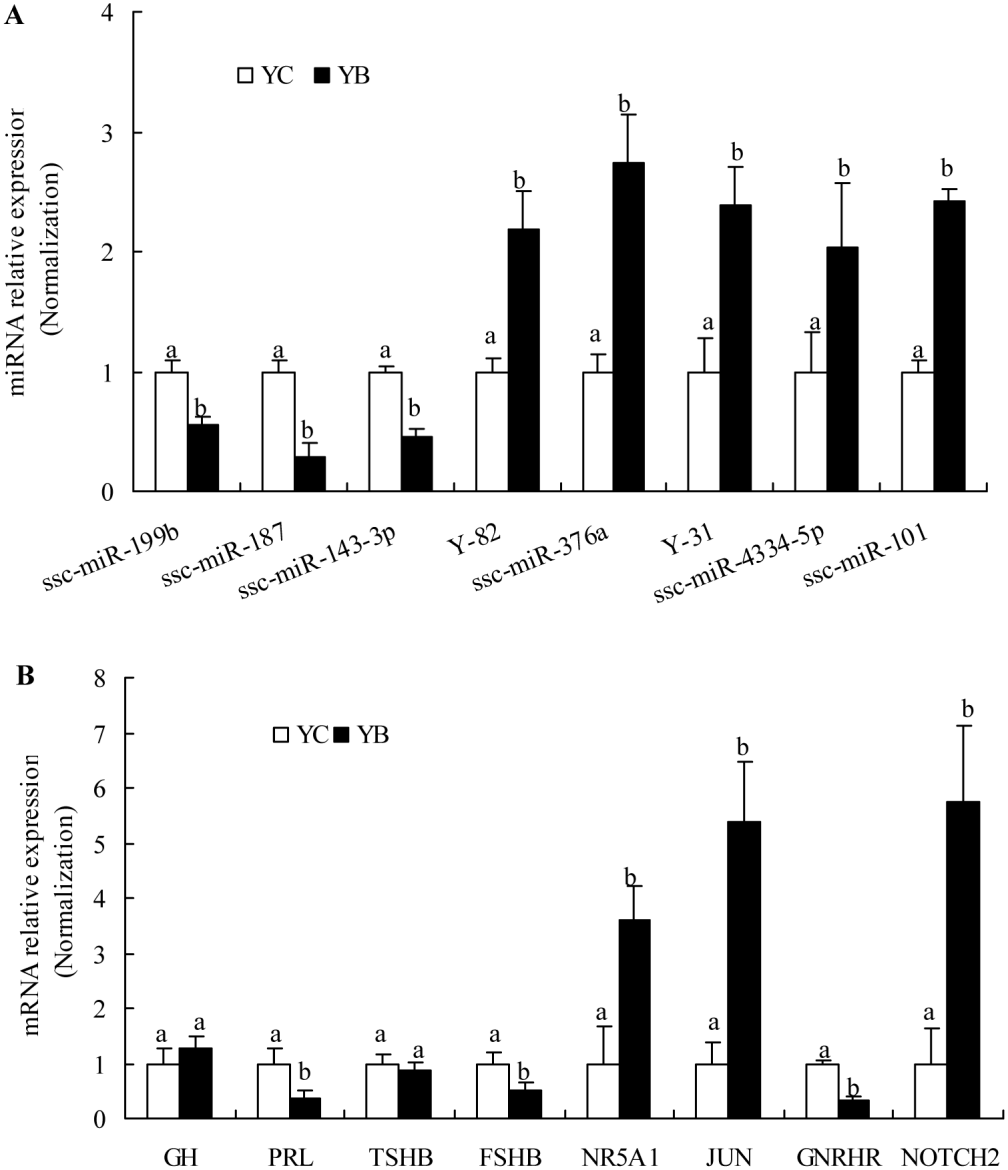
qRT–PCR validations of miRNAs and mRNAs (transcripts). (A) Eight differentially expressed miRNAs. (B) Five differentially expressed and three important transcripts in the pituitary. Data in columns are means ± SD. The expression abundance of Landrace (YC) was normalized to 1. Statistical significance was determined by Student's t test, p<0.05 was considered significant.; the panels with different letters were considered statistically significant (p < 0.05).

Filtering by |fold-change| ≥ 2 and p ≤ 0.01, a total of 41 differentially expressed miRNAs were identified between Bama minipigs and Landrace pigs (YB versus YC), comprising 32 up-regulated miRNAs and 9 down-regulated miRNAs (Table 1 and cluster analysis was shown in Fig 1E). Interestingly, some miRNA families were differentially expressed in Bama minipigs and Landrace pigs. For example, the miR-133 family (ssc-miR-133b, ssc-miR-133a-3p) as well as the let-7 family (ssc-miR-98, ssc-let-7e) was up-regulated in Bama minipigs; in the miR-1/206 family, ssc-miR-1 was up-regulated whilst ssc-miR-206 was down-regulated in Bama minipigs. To validate these differentially expressed miRNAs obtained through the miRNA arrays, eight miRNAs (miR-199b, miR-187, miR-143-3p, Y-82, miR-376a, Y-31, miR-4334-5p, and miR-101) were selected for quantitative reverse transcription–PCR (qRT–PCR) analysis. As shown in Fig 2A, the miRNA expression patterns in the two breeds correspond to the results revealed by miRNA microarray.

**Table 1:** Differentially expressed miRNAs between Bama minipigs (YB) and Landrace (YC).

Reporter Name	Log2(YB/YC)	p-value	Reporter Name	Log2(YB/YC)	p-value
**Up-regulated miRNAs**					
ssc-miR-1	4.08	3.30E-03	ssc-miR-338	1.41	1.78E-06
ssc-miR-325	3.21	4.53E-03	ssc-miR-101	1.36	5.77E-15
Y-33	2.94	5.72E-03	ssc-miR-19b	1.34	0.00E+00
ssc-miR-133b	2.44	1.48E-11	Y-47	1.34	5.03E-04
Y-82	2.32	1.50E-13	Y-69	1.30	1.07E-05
ssc-miR-133a-3p	2.23	1.52E-08	Y-24	1.27	2.89E-15
Y-12	2.10	5.97E-12	ssc-miR-135	1.26	2.45E-06
ssc-miR-193a-3p	2.04	4.15E-07	ssc-miR-628	1.26	4.46E-06
ssc-miR-95	1.87	1.31E-08	Y-31	1.25	4.62E-11
ssc-miR-98	1.83	1.10E-10	Y-44	1.24	8.37E-11
Y-22	1.72	1.27E-03	Y-56	1.21	4.92E-10
ssc-miR-4331	1.70	8.26E-06	ssc-miR-149	1.15	3.18E-11
ssc-miR-4334-5p	1.64	8.86E-10	ssc-miR-365-3p	1.15	2.34E-04
Y-23	1.59	4.24E-14	ssc-let-7e	1.09	6.40E-09
ssc-miR-376a	1.50	1.73E-09	ssc-miR-28-5p	1.02	1.72E-03
Y-30	1.48	6.77E-09	Y-67	1.01	8.65E-11
**Down-regulated miRNAs**					
ssc-miR-224	-3.30	1.82E-11	ssc-miR-183	-1.25	4.08E-11
ssc-miR-202	-2.18	3.40E-06	ssc-miR-206	-1.25	4.16E-10
ssc-miR-296	-1.77	1.80E-08	ssc-miR-199a*	-1.04	6.22E-15
ssc-miR-187	-1.30	4.38E-04	ssc-miR-143-3p	-1.01	2.18E-12
ssc-miR-199b	-1.28	2.44E-15			

YB, YC represent Bama minipigs and Landrace pigs, respectively. miRNAs underlined represents this differential miRNAs is p ≤ 0.01 and the higher signal≥500.

### Gene-expression analysis

**Fig 3:**
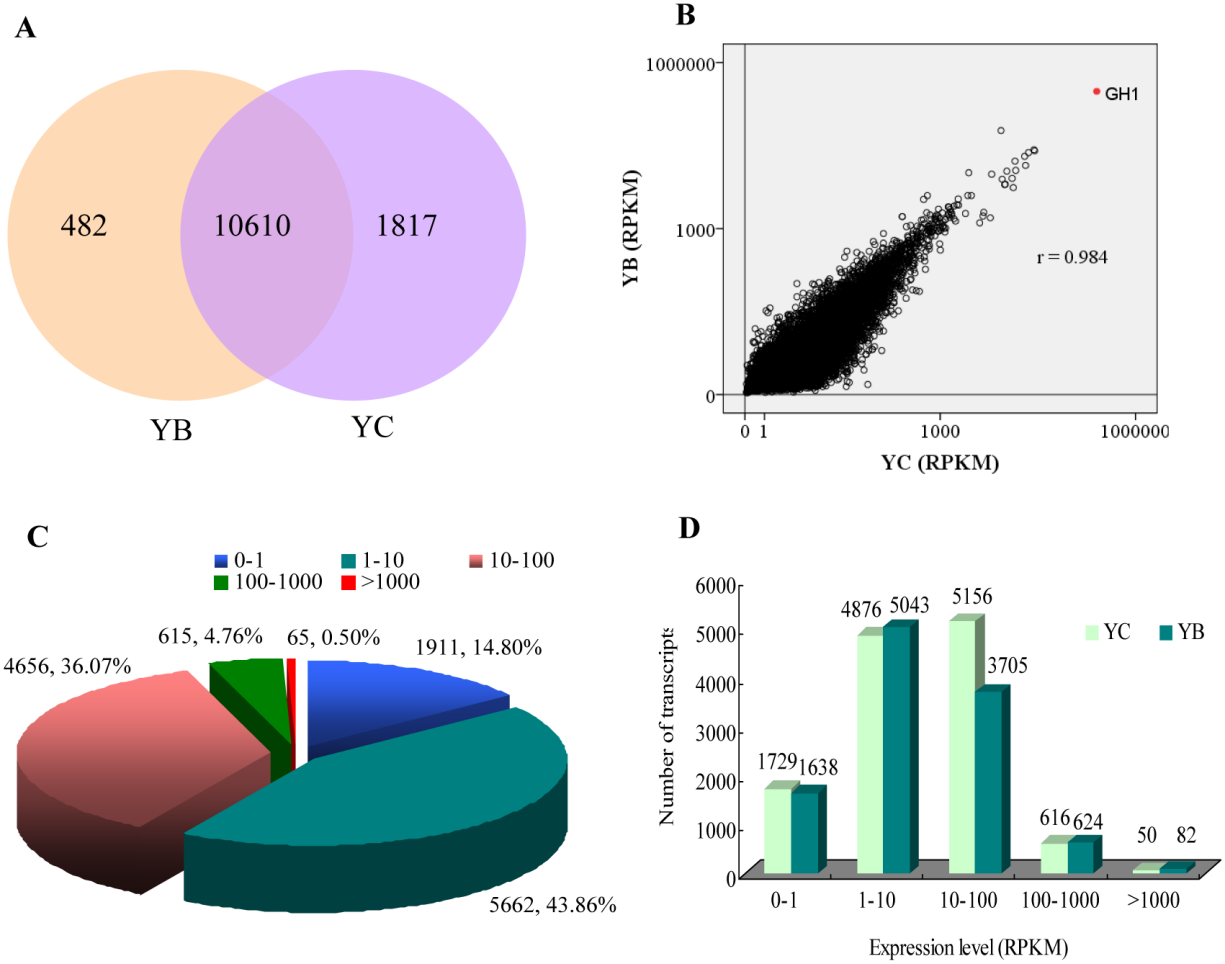
mRNA (transcripts) expression and distribution. (A) mRNA numbers of the two pig breeds. (B) Scatter plots of mRNA expression; *r* represents the correlation between Bama minipigs and Landrace pigs and the red dot GH1 was the most abundant transcript in both pig breeds. (C) The average expression level and proportion of mRNAs in the anterior pituitary. (D) The mRNA numbers of the two pig breeds at different expression levels. YB and YC indicate Bama minipigs and Landrace pigs, respectively.

In this study, the anterior pituitary mRNA expressions was analysed using mRNA-seq, taking into account only those genes with average RPKM greater than zero; 12,909 porcine transcripts were detected in the two pig breeds (Fig 3A). Correlation analysis of the overall gene-expression levels among the two breeds indicated that the anterior pituitary transcriptomes of the two breeds was quite similar (Pearson’s correlation coefficient, r = 0.984 in Bama minipigs and Landrace pigs) (Fig 3B); 10,610 transcripts were commonly expressed among the breeds, and 482 and 1,817 were breed-specific in Bama minipigs and Landrace pigs respectively (S4 Table). Interestingly, most were expressed at a low number of reads, suggesting that these transcripts are generally expressed at a low level in the anterior pituitary and are hard to detect. Such transcripts were often filtered out and excluded from further differentially expressed transcript analysis.

Gene-expression distribution (average RPKM) revealed that 58.66% of transcripts were expressed at less than 10 RPKM, 36.07% at 10–100 RPKM, around 4.76% were from 100 to 1,000 RPKM, and only 0.50% transcripts were more than 1,000 RPKM (Fig 3C). Interestingly, a large difference was also observed in that 5,156 and 3,705 transcripts expressed at 10–100 RPKM in Landrace and Bama minipigs, respectively (Fig 3D).

Of these transcripts, *GH1* mRNA was the most abundant transcript of the pig anterior pituitary, which accounted for 30.09% (average RPKM/total RPKM) of all porcine transcript expression in this organ (31.17% and 29.05% in Bama minipigs and Landrace pigs, respectively). Furthermore, pituitary-specific hormone transcripts (i.e. *PRL*, *CGA*, *LHB*, *FSHB*, and *POMC*) as well as other endocrine-related transcripts (i.e. *CHGB*, *CLU*, *CALML4*, *CALR*, *GABARAP*, *NNAT*, and *SCG5*) are highly expressed in anterior pituitary, which are listed on the top100 transcripts, which is quite similar with a previous study[17]. KEGG analysis showed that the top100 transcripts enriched in the ribosome, Parkinson's disease, and oxidative phosphorylation pathway, suggesting that these transcripts may be critical for maintaining the basic function of the pituitary.

### Differential gene-expression analysis

By filtering data by |fold-change| ≥ 2 and FDR corrected p-value ≤ 0.001, we identified 2,254 transcripts that were differentially expressed in YB versus YC, which include 446 up-regulated and 1,808 down-regulated transcripts (S5 Table) and the expression of *GH*, *PRL*,*TSHB*,*FSHB*,*NR5A1*,*JUN*,*GNRHR*,*NOTCH2* was selected for validation by qRT-PCR(see Fig 2B).

To further understand the possible biological pathways affected by differentially expressed genes, we analysed the potential KEGG pathways enriched in differentially expressed transcript between two breeds; 32 KEGG pathways (p < 0.05) were identified (Table 2 and S6 Table) as being involved in metabolism, disease, cell communication, and endocrine regulation, all of which may contribute to the difference between Bama minipigs and Landrace pigs. It is worthwhile to note that eight pathways were closely related to pituitary endocrine functions, including the neurotrophin signalling pathway, the phosphatidylinositol signalling system, the GnRH signalling pathway, inositol phosphate metabolism, the VEGF signalling pathway, the mTOR signalling pathway, axon guidance, and long-term potentiation. In particular, the phosphatidylinositol signalling system and inositol phosphate metabolism are two pathways responsible for intracellular inositol 1,4,5-triphosphate (IP3) metabolism and signal transduction that directly participated in the anterior pituitary GH secretion[7]. Interestingly, the majority of transcripts (29/36) in these two pathways were significantly down-regulated in Bama minipigs, which might attenuate the pituitary GH-secretion-signalling response to extracellular stimulus (such as GHRH, SS).

**Table 2:** KEGG pathways analysis of differentially expressed mRNAs.

No.	Pathway	observed	P-value
1	ssc04141:Protein processing in endoplasmic reticulum	69	6.83E-09
2	ssc04510:Focal adhesion	90	2.17E-06
3	ssc04530:Tight junction	78	3.76E-05
4	ssc04722:Neurotrophin signaling pathway	50	3.21E-04
5	ssc04512:ECM-receptor interaction	44	3.83E-04
6	ssc04670:Leukocyte transendothelial migration	51	5.67E-04
7	ssc05222:Small cell lung cancer	32	3.78E-03
8	ssc04070:Phosphatidylinositol signaling system	35	4.90E-03
9	ssc04912:GnRH signaling pathway	34	6.50E-03
10	ssc04962:Vasopressin-regulated water reabsorption	16	7.54E-03
11	ssc04062:Chemokine signaling pathway	49	8.87E-03
12	ssc04720:Long-term potentiation	28	9.68E-03
13	ssc05200:Pathways in cancer	88	9.89E-03
14	ssc04144:Endocytosis	71	9.94E-03
15	ssc04810:Regulation of actin cytoskeleton	78	1.02E-02
16	ssc05130:Pathogenic Escherichia coli infection	36	1.62E-02
17	ssc00562:Inositol phosphate metabolism	22	2.01E-02
18	ssc05412:Arrhythmogenic right ventricular cardiomyopathy (ARVC)	29	2.06E-02
19	ssc05110:Vibrio cholerae infection	24	2.07E-02
20	ssc04520:Adherens junction	33	2.20E-02
21	ssc04370:VEGF signaling pathway	27	2.58E-02
22	ssc00780:Biotin metabolism	2	2.70E-02
23	ssc04150:mTOR signaling pathway	20	2.87E-02
24	ssc03060:Protein export	8	3.02E-02
25	ssc00512:Mucin type O-Glycan biosynthesis	7	3.12E-02
26	ssc00510:N-Glycan biosynthesis	15	3.36E-02
27	ssc05131:Shigellosis	29	3.57E-02
28	ssc05211:Renal cell carcinoma	20	3.58E-02
29	ssc00030:Pentose phosphate pathway	9	4.26E-02
30	ssc04360:Axon guidance	44	4.33E-02
31	ssc00310:Lysine degradation	20	4.68E-02
32	ssc05100:Bacterial invasion of epithelial cells	30	4.88E-02

### Analysis of GH-regulation-related genes

Due to the importance of GH in animal growth regulation and according to the previous reviews[6, 7], we focused on genes associated with GH-regulation-related genes. We summarized the genes mediating in the regulation of GH (Table 3). Surprisingly, GH gene *GH1* and the pituitary-specific transcript factor *POU1F1* (*Pit-1*) were not significantly changed, but we observed that *Pit-1*, as well as transcription factor *SP1*, the gene assisting Pit-1 to bind to the *GH* promoter[5], showed a down-regulated tendency (over 2-fold) in Bama minipigs (*p*-values for *Pit-1* and *SP1* were 0.17 and 0.005, respectively; see S4 Table). Notably, apart from the IP3 signalling revealed by the KEGG pathway, we also observed that transcripts in cAMP signalling differed between the two breeds, including G-protein (*GNAQ*, *GNAI3*, *GNAZ*, *GNAI1*, *GNAS*), adenylate cyclase (*ADCY6*, *ADCY8*), and CREB family members (*CREB3L1*, *CREB3L2*, *ATF2*, *ATF6*) that were all significantly down-regulated in Bama minipigs, which may attenuate the pituitary GH-secretion-signalling response to extracellular stimulus. In addition, we also observed that GH-secretion-related genes *GAL*, *YKT6*, *SNAP23*, and *SNAP91* as well as other GH regulators *RARA*, *THRA*, and *ACVR1* were significantly changed between Bama minipigs and Landrace pigs.

**Table 3:** Differentially expressed genes associated with growth hormone regulation.

Gene name	Description	Log2(YB/YC)	FDR
**cAMP signaling (G-proteins, adenylate cyclase and CREB)**			
GNAQ	guanine nucleotide-binding protein G(q) subunit alpha	-2.01	5.60E-04
GNAI3	guanine nucleotide binding protein, alpha inhibiting 3	-1.57	1.50E-09
GNAZ	guanine nucleotide-binding protein G(z) subunit alpha-like	-1.98	2.89E-05
GNAI1	guanine nucleotide binding protein, alpha inhibiting 1	-1.83	1.32E-08
GNAS	GNAS complex locus	1.76	1.42E-07
ADCY6	adenylate cyclase 6	-1.99	1.87E-12
ADCY8	adenylate cyclase 8 (brain)	-1.22	5.39E-05
CREB3L1	cAMP responsive element binding protein 3-like 1	-2.82	2.68E-23
CREB3L2	cyclic AMP-responsive element-binding protein 3-like protein 2-like	-1.19	3.34E-04
ATF2	cyclic AMP-dependent transcription factor ATF-2-like	-2.43	1.06E-08
ATF6	cyclic AMP-dependent transcription factor ATF-6 alpha-like	-1.91	6.16E-07
**Secretion-related genes**			
GAL	galanin/GMAP prepropeptide	-4.17	9.16E-05
YKT6	YKT6 v-SNARE homolog (S. cerevisiae)	-1.17	6.19E-07
SNAP23	synaptosomal-associated protein, 23kDa	-2.18	5.51E-06
SNAP91	synaptosomal-associated protein, 91kDa homolog (mouse)	-2.21	4.00E-08
**Receptors**			
RARA	retinoic acid receptor alpha isoform 2	-1.93	4.25E-04
THRA	Thyroid hormone receptor alpha	1.42	6.26E-06
ACVR1	PREDICTED: activin receptor type-1	-1.88	2.75E-06

YB, YC represent Bama minipigs and Landrace pigs, respectively.

### miRNA–mRNA interaction analysis

**Fig 4:**
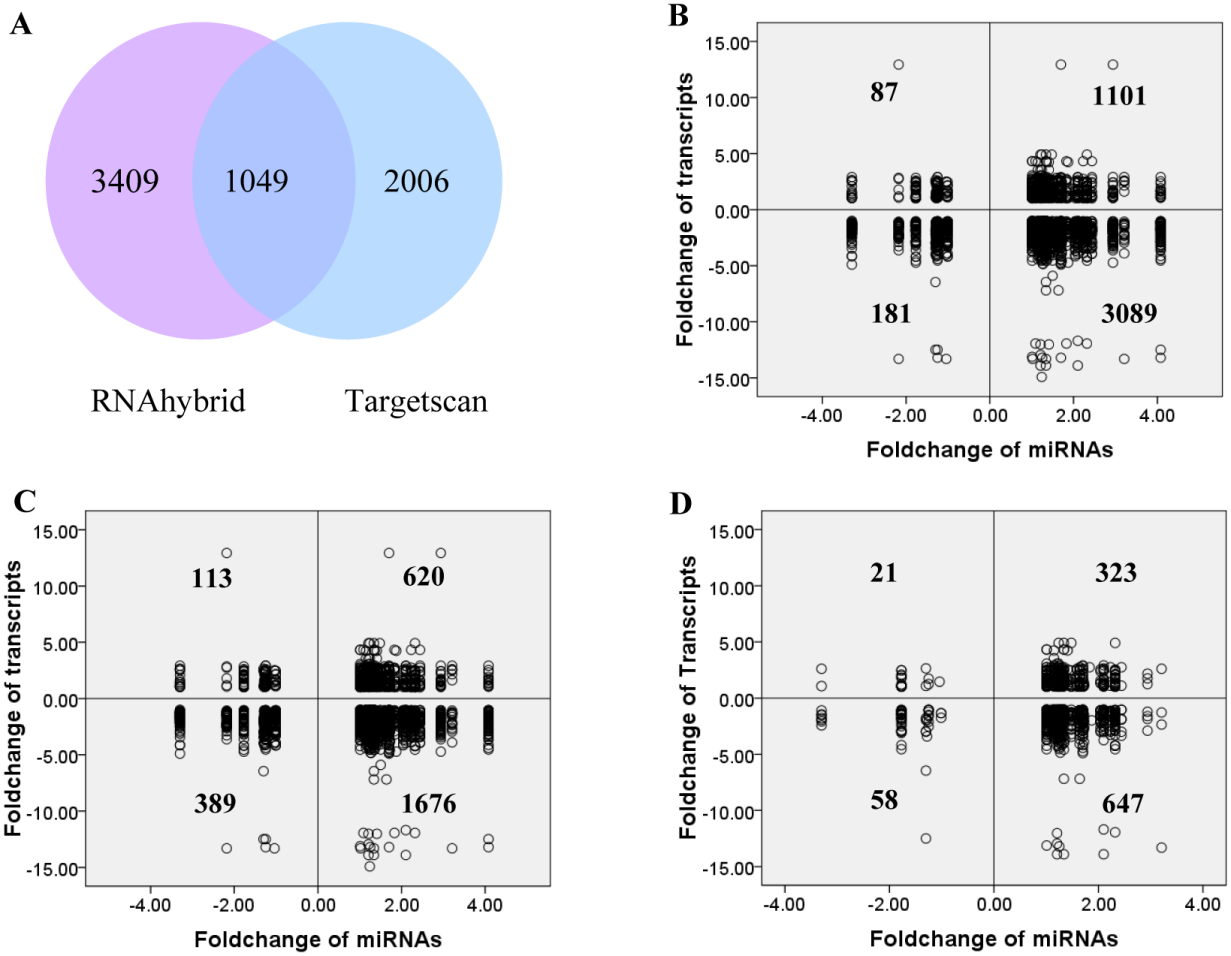
miRNA–mRNA interaction analysis by RNAhybrid and TargetScan. (A) The number of predicted miRNA–mRNA pairs by two algorithms. (B) miRNA–mRNA pair distribution in the RNAhybrid prediction. (C) miRNA–mRNA pair distribution in the TargetScan prediction. (D) The overlapping miRNA–mRNA pair distributions. The number in each quadrant represents the number of predicted miRNA–mRNA pairs. Dots in the second and fourth quadrants are negatively correlated miRNA–mRNA interaction pairs.

miRNAs regulate gene expression at the post-transcriptional level. Most studies believe that miRNAs have little influence on mRNA abundance. Interestingly, we observed that more up-regulated miRNAs (32 up-regulated and 9 down-regulated) in YB versus YC comparison coincided with more down-regulated transcripts (1,832 down-regulated and 498 up-regulated). We speculated that miRNAs may have a substantial effect on mRNA abundance. To further understand the possible relationship between miRNAs and mRNAs (transcripts), target predictions were performed for all differentially expressed miRNAs (41 differentially expressed miRNAs) and differentially expressed transcripts (1,341 of 2,254 transcript 3-UTRs were annotated in the Ensemble database) by RNAhybrid algorithm. A total of 4,458 possible miRNA–mRNA interaction pairs were predicted (Fig 4A). Interestingly, the majority of miRNA–mRNA interactions (71.33%) present a negatively correlated expression pattern (Fig 4B), referring to up-regulated miRNAs and down-regulated transcript targets, or down-regulated miRNAs and up-regulated transcript targets. Furthermore, these results were also confirmed using the TargetScan algorithm (63.94% negatively correlated) (Fig 4C) and the overlapping miRNA–mRNA pairs between the RNAhybrid algorithm and the TargetScan algorithm (63.68%) (Fig 4D and S7 Table). Our results suggest that miRNAs might have an important negative regulatory effect on mRNA abundance.

**Fig 5:**
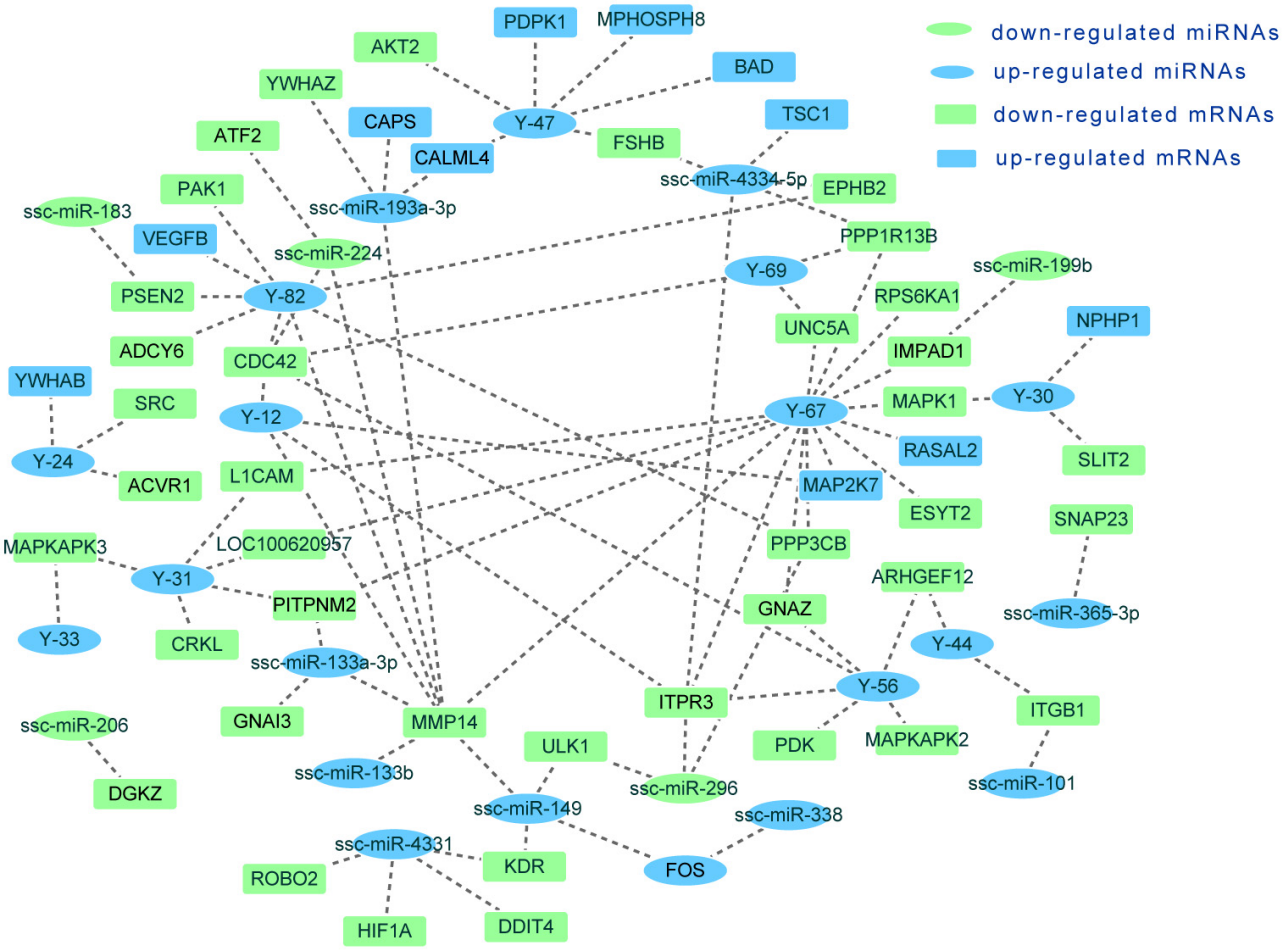
The potential network of differentially expressed miRNAs and mRNAs that are involved in animal growth regulation. Target pairs were predicted by RNAhybrid and TargetScan.

To further explain the possible mechanism involved in growth differences between Bama minipigs and Landrace pigs, miRNA–mRNA analysis narrowed down to key genes and pituitary endocrine-related pathways identified by KEGG analysis. Using the RNAhybrid and TargetScan algorithms, we summarized the potential target relationship between anterior pituitary endocrine-related transcripts and differentially expressed miRNAs, which are more likely to take part in animal growth regulation, especially the miRNA–mRNA pairs involved in GH synthesis and secretion signalling (cAMP and IP3 signalling). For instance, Y-82 targets *ADCY6* that is involved in cAMP signalling; miR-4334-5p, Y-67, and Y-12 potentially target *ITPR3* of IP3 signalling; ssc-miR-365-3p potentially target the GH-secretion-related molecule *SNAP23*. These data suggest that miRNA, mRNA, and signalling may form a complicated network regulating animal postnatal growth, as shown in Fig 5.

### miRNA-mRNA interaction validated by luciferase assay

**Fig 6:**
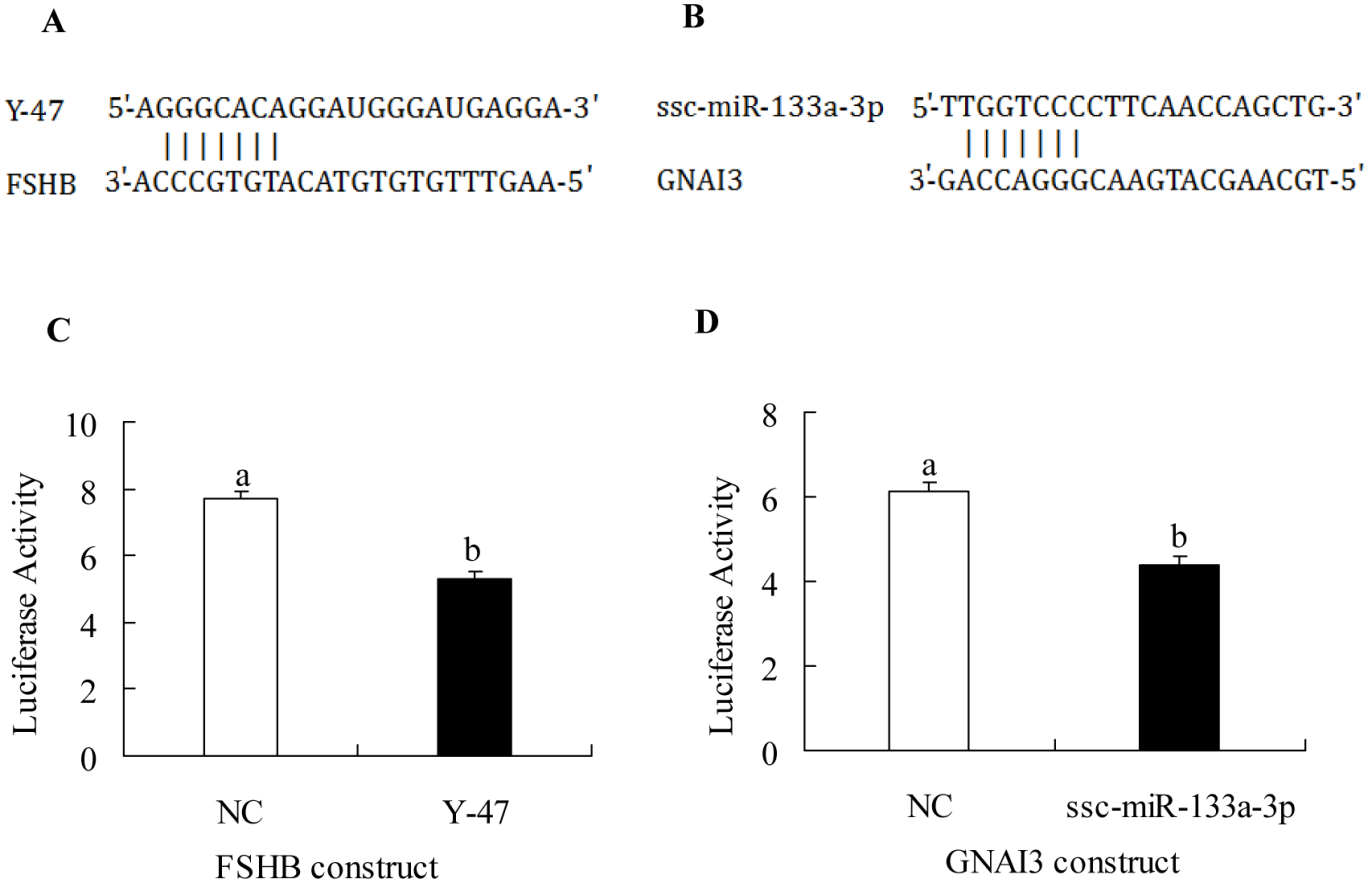
miRNA-mRNA interaction conformed by luciferase assay. (A) The second structure of Y-47(custom-designed miRNA) and its potential target FSHB (for seed sequence only). (B) The second structure of ssc-miR-133a-3p and its potential target GNAI3 (for seed sequence only). (C)Y-47 mimics significantly decreased FSHB construct luciferase activity (relative, firefly luciferase activity/Renilla luciferase activity). (D) ssc-miR-133a-3p mimics significantly decreased GNAI3 construct luciferase activity (relative, firefly luciferase activity/Renilla luciferase activity). Statistical significance was determined by ANOVA, followed by Tukey’s multiple comparisons test, the panels with different letter were considered statistically significant (p<0.05).

To validate the potential network involved in animal growth regulation, two miRNA-mRNA interactions (Y-47 potentially target FSHB, Fig 6A; ssc-miR-133a-3p potentially target GNAI3, Fig 6B) were analyzed by luciferase assay. Dual-luciferase reporter recombinant FSHB 3′-UTR plasmid and GNAI3 3′-UTR plasmid were constructed, respectively. Recombinant FSHB 3′-UTR plasmid (FSHB construct) and recombinant GNAI3 3′-UTR plasmid (GNAI3 construct) were transfected with their corresponding miRNA mimics, respectively. Luciferase activity measurement showed Y-47 significantly decreased FSHB construct activity by 31.48% (p<0.001, Fig 6C), while ssc-miR-133a-3p significantly decreased GNAI3 construct activity by 28.41% (p<0.001, Fig 6D). These data suggest the miRNAs negatively regulate mRNAs and they may form a complicated miRNA-mRNAs network controlling animal postnatal growth.

## Discussion

The anterior pituitary is a small but powerful endocrine organ implicated in animal postnatal growth via GH. The present study carried out a preliminary investigation of miRNA and mRNA expression in two breeds of pigs (Bama minipigs and Landrace pigs) that have extreme growth differences, so that potential regulators of animal postnatal growth from the anterior pituitary perspective could be identified. In the present study, we simultaneously analyze miRNA and mRNA expression profiles of normal anterior pituitaries by combined high-throughput technologies (miRNA microarrays and mRNA-Seq).

### mRNA and miRNA expression

In the present study, a total of 222 miRNAs and 12,909 transcripts were detected in Bama minipig and Landrace pig anterior pituitary; Relative global expression of both miRNAs and mRNAs were found to be are quite similar in the two breeds (*r* > 0.97 in both miRNA and mRNA expressions). Although breed-specific differences in miRNAs/mRNAs occurred, most of them occurred at a low transcript number in one breed while remained undetectable in the other breed. It is therefore reasonable to propose that these miRNAs/mRNAs are expressed in the pig anterior pituitaries, and are filtered out in differential expression comparison.

A total of 20 miRNAs were highly expressed with a signal value of over 10,000, most of which have been reported and their functions have been well studied. For example, miR-7 is the most abundant miRNA in both pig breeds, which was consistent with our previous study[18] and studies in mouse[24]; it was reported that miR-375 mediated in POMC regulation by targeting mitogen-activated protein kinase 8[25] and miR-200 mediated in female fertility, regulating LH secretion by targeting *ZEB1*[26]. Interestingly, these highly expressed miRNAs are relatively stable in Bama minipigs and Landrace pigs, suggesting that they may be crucial in maintaining the normal function of the pituitary; aberrant expression of miRNAs will lead to anterior pituitary dysfunction such as pituitary adenoma. It was reported that anterior pituitary-enriched miR-21 was down-regulated in ACTH-secreting pituitary tumours[27].

Similarly to previous pituitary transcriptome studies[17], we also observed hormone transcripts (*GH1*, *CGA*, *PRL*, *CGA*, *LHB*, *FSHB*, and *POMC*) being expressed at extremely high levels (list in top100 transcripts). However, this high level expression is not surprising as these hormone transcripts are essential for the tissue-specific function of the pituitary gland. Interestingly, upon closer examination of their mRNA abundance, this corresponds perfectly with their cell proportion in the anterior pituitary; for example, somatotrophs are the most common cell type (up to 50%) in the anterior pituitary[28] and *GH1* were the most abundant transcripts in our study. In addition, we also detected endocrine-related *CHGB*, *NNAT*, *CLU*, and *CALR*, and other non-specific genes (such as *RPS* family and *RPL* family) were listed in the top 100 genes. As described in our results, they are involved in the ribosome and oxidative phosphorylation pathways, which are necessary for basic pituitary function. However, we observed that several important endocrine-related transcripts such as *SCG5*, *GABARAP*, and *CALML4* were also present in our top100 genes while they were expressed at a relatively low level in the previous study[17]. One possible reason is that the previous study collected the whole pituitary (anterior pituitary and posterior pituitary) for analysis whereas we focused only on the anterior pituitary region.

### Potential interaction between miRNAs and mRNAs

It was firmly believed that miRNAs are small RNAs that regulate gene expression at the post-transcriptional level. But there is still controversy regarding the effect of animal miRNAs on their target mRNAs’ abundance. miRNAs were thought to repress protein output only by translation repression, with little or no influence on mRNA levels. However, accumulating evidence shows that miRNAs eventually down-regulate their target mRNAs’ abundance, either by mRNA destabilization (such as deadenylation)[29–31]or by miRNA-mediated Argonaute-catalysed mRNA cleavage[32]. In the present study, miRNA and mRNA expressions were simultaneously analysed; target prediction showed a high proportion (about 70%) of differentially expressed miRNA–mRNA pairs presented negatively correlated expression patterns by two different prediction algorithms and most of them are represented by up-regulated miRNAs and down-regulated mRNAs. Our results support the hypothesis that mammalian miRNAs may predominantly act to decrease target mRNA levels[29]. For the remaining miRNA–mRNA pairs (about 30%) that did not show negatively correlated expression patterns, several explanations are possible: (a) miRNAs may regulate mRNAs by targeting other regions (such as 5′-UTR and CDS)[33, 34]; (b) some targets of miRNAs were repressed without detectable changes in mRNA levels[35, 36]; (c) miRNAs’ actions resulted in the increase in target mRNA abundance[37]; and (d) miRNAs alter mRNA abundance indirectly[38].

### The potential mRNAs/miRNAs/pathways mediating in animal postnatal growth

Dramatic differences in growth traits were observed in Bama minipigs and Landrace pigs. Although animal postnatal growth is complicated, and is influenced by many factors across the whole body, the anterior pituitary remains the most important organ for regulating animal postnatal growth by synthesizing, storing, and secreting GH, which is controlled by thousands of molecules at multiple levels (such as transcription, post-transcription, translation); in the present study, we compared the anterior pituitary miRNA and mRNA expressions at the whole-genome level.

By comparing mRNA expressions at the whole-genome level, a variety of genes were identified; unexpectedly, *GH1*, transcript factors such as *POU1F1*, *SP-1*, as well as classical receptors including *GHRHR* and *SSTRs* were not significantly changed between Bama minipigs and Landrace pigs, but it seemed as if the transcriptional difference between Bama minipigs and Landrace pigs may be represented by GH-secretion signalling rather than GH synthesis itself, which was revealed by the signalling pathways and differentially expressed mRNAs. It was well established that many GH regulators (such as GHRH, SS) can regulate GH through cAMP signalling and IP3 signalling[7, 39]. The cAMP signalling cascade consists of G-proteins, Adenylate Cyclase (AC), cAMP, PKA, and CREB, and any change in each step may affect pituitary GH. The present study shows that differentially expressed genes occurred in almost all steps of cAMP signalling. In addition, IP3 signalling was also important in GH regulation. KEGG pathways showed that IP3 changes are present not only in inositol phosphate metabolism, but also in the phosphatidylinositol signalling system. More importantly, most differentially expressed genes in both cAMP signalling and IP3 signalling are significantly down-regulated in Bama minipigs, which may attenuate the effect of the upstream regulator on GH synthesis and/or secretion. Notably, our study also showed that GH-secretion-related genes were significantly down-regulated in Bama minipigs, including GH release regulator Galamin (*GAL*)[40], as well as SNARE family members (*YKT6*, *SNAP23*, *and SNAP91*) that play an important role in transporting hormone-secreting vesicles to other cells or tissues[7, 41]. Taken together, down-regulation of these genes may lead to a decrease in signal transduction capability and sensitivity in response to GH regulator stimuli, which may partly explain the lower growth performance of Bama minipigs, although their exact mechanism needs further investigation.

Differential expression of miRNAs may also contribute to different GH regulation; in our study, 32 up-regulated miRNAs and 9 down-regulated miRNAs were identified in Bama minipigs versus Landrace pigs. It has been well demonstrated that let-7, miR-193-3p, and miR-195 were down-regulated in GH-secreting pituitary adenomas that accompanied excessive GH secretion[42, 43] and our previous study demonstrated that over-expression of ssc-let-7c in porcine pituitary cells leads to a decrease in GH secretion[44]. In the present study, we observed that two members of the let-7 family (let-7e and ssc-miR-98) as well as miR-193-3p and miR-195 were significantly up-regulated in Bama minipigs. Therefore, up-regulation of these miRNAs in Bama minipigs may possibly decrease GH secretion in the pituitary. In addition, there were other miRNAs such as miR-365, miR-183, miR-149, miR-224, and miR-199b that were frequently aberrantly expressed in GH-secreting or other pituitary adenomas[45, 46], although their detailed function is still unknown. They may regulate GH by other pathways or participate in other functions of the pituitary.

Moreover, the observed differences between Bama minipigs and Landrace pigs may be regulated by a combined effect of miRNAs and mRNAs. miRNA–mRNA interaction prediction showed a network among miRNAs, mRNAs, and signalling pathways that potentially regulates GH. According to luciferase assay results, Y-47 target FSHB, and ssc-miR-133a-3p target GNAI3, both of which may play important roles in GH regulation. Overall, differentially expressed miRNAs may exert their effect on the pituitary by a single molecule or multiple regulators/pathways including miRNAs and mRNAs.

### Conclusions

In conclusion, using a whole-genome comparison of mRNA and miRNA expression profiles, 41 miRNAs and 2,254 transcripts were differentially expressed in Bama minipigs and Landrace pigs. Target prediction analysis showed most differentially expressed miRNAs-differentially expressed mRNAs pairs are negatively correlated. Additionally, a variety of pituitary endocrine-related transcripts and pathways were significantly changed, including cAMP and inositol 1, 4, 5-triphosphate (IP3) signalling. Bioinformatics analysis indicated these transcripts and differentially expressed miRNAs potentially form a miRNAs-mRNAs network regulating GH secretion, as validated by luciferase assay (Y-47 target FSHB, and ssc-miR-133a-3p target GNAI3). The miRNAs and mRNAs revealed in this study will be helpful in understanding the possible mechanism involved in animal postnatal growth, although more detailed studies remain to be elucidated in the future.

## Supporting Information

S1 Table 100 designed miRNAs probes information. (XLS)

S2 Table Primers for qRT-PCR. (DOC)

S3 Table All miRNAs expression. (XLS)

S4 Table All detectable transcripts. (XLS)

S5 Table Differentially expressed transcripts. (XLS)

S6 Table KEGG pathway analysis. (XLS)

S7 Table miRNAs-mRNAs interaction analysis. (XLS)

S1 Text DNA oligos for recombinant 3′-UTR pmirGLO vector. (DOC)
