# Supplementary material for: Comparative Anterior Pituitary miRNA and mRNA Expression Profiles of Bama Minipigs and Landrace Pigs Reveal Potential Molecular Network Involved in Animal Postnatal Growth
Source: PLoS One. 2015 Jul 2;10(7):e0131987. doi: 10.1371/journal.pone.0131987 (PMC4489742; doi:10.1371/journal.pone.0131987)
Supplement: S2 Table — (DOC) [file pone.0131987.s002.doc]

**TableS2. Primers information of qRT-PCR validation of miRNAs and genes**

| Accession | Gene/miRNA | Primer Sequence(5' to 3') | | Products  (bp) | Tm(℃) |
| --- | --- | --- | --- | --- | --- |
| MIMAT0017339 | ssc-miR-199b | CCCAGTGTTTAGACTATCTGTT | | 79 | 60 |
| MIMAT0020587 | ssc-miR-187 | TCGTGTCTTGTGTTGCAGCC | | 79 | 58 |
| MIMAT0013879 | ssc-miR-143-3p | TGAGATGAAGCACTGTAGCTC | | 78 | 59 |
|  | Y-82 | TAGGGGGCAGGAGCCGGAGCC | | 82 | 62 |
| MIMAT0015716 | ssc-miR-376a | ATCATAGAGGAAAATCCACGT | | 78 | 60 |
| MIMAT0013873 | ssc-miR-30e-3p | CTTTCAGTCGGATGTTTACAGC | | 79 | 60 |
|  | Y-31 | GAGCTGGAGAAGGAGGTGG | | 78 | 58 |
| MIMAT0017966 | ssc-mir-4334-5p | CCCTGGAGTGACGGGGGTG | | 76 | 58 |
| MIMAT0010185 | ssc-miR-101 | TACAGTACUGTGAUAACTGAA | | 78 | 60 |
| / | U6 | Forward | CTCACTTCGGCAGCACATA | 94 | 58 |
|  |  | Reverse | AACTCTTCACGATTTTGTCTGTC |  |  |
| NM_213869.1 | GH1 | Forward | CACCAACTGGCTGCCGACACCTA | 150 | 56 |
|  |  | Reverse | CGATCTCTGCTGGGCCTCGTCC |  |  |
| NM_214035.2 | GHRHR | Forward | CTGGTGGATCATCAAAGGG | 161 | 60 |
|  |  | Reverse | TGAGGAGAAGGGTTGACTTG |  |  |
|  |  | Reverse | GTGGCCCATGACGTACTTC |  |  |
| NM_214368.2 | TSHB | Forward | TATGACACGGGATTTCAATG | 111 | 58 |
|  |  | Reverse | GGGCATCCTGGTATTTCTAC |  |  |
| NM_213926.1 | PRL | Forward | CAGAGGGTTCATTACCAAGGC | 151 | 58 |
|  |  | Reverse | GTGATACAGCGGGTCATTCC |  |  |
|  |  | Reverse | GGAATTGTTGGCTAACAGAC |  |  |
| XM_003481477.1 | NOTCH2 | Forward | CCTGCATTGACCGTGTA | 88 | 60 |
|  |  | Reverse | ACTGGAAGCGATTGACT |  |  |
| NM_214179.1 | NR5A1 | Forward | GAGGAGGTTTGGATTCCCC | 90 | 60 |
|  |  | Reverse | TTCGCAGACTCTTGTTAGCC |  |  |
| NM_214273.1 | GNRHR | Forward | TCACCTTTAGCTGCCTCTTC | 94 | 58 |
|  |  | Reverse | CTCGTGGTATGTTGTTCTTGG |  |  |
| NM_213875.1 | FSHB | Forward | CCATCTCCCAATCTGTCTC | 177 | 58 |
|  |  | Reverse | GCATTTAGTCCTTTCACCC |  |  |
| AY550069.1 | β-actin | Forward | CCAGCACCATGAAGATCAAGATC | 110 | 60 |
|  |  | Reverse | ACATCTGCTGGAAGGTGGACA |  |  |
