# Supplementary material for: Comparative Anterior Pituitary miRNA and mRNA Expression Profiles of Bama Minipigs and Landrace Pigs Reveal Potential Molecular Network Involved in Animal Postnatal Growth
Source: PLoS One. 2015 Jul 2;10(7):e0131987. doi: 10.1371/journal.pone.0131987 (PMC4489742; doi:10.1371/journal.pone.0131987)
Supplement: S1 Text — (DOC) [file pone.0131987.s008.doc]

**S1 Text. DNA oligos for recombinant 3’-UTR pmirGLO vector**

**FSHB 3’-UTR sense sequence (partial):**

**5’-TCGAG**AGAGCAGTGGACATTTCATGCTTCCTACCCTTGTCTGAAGGACCAAGACGTCCAAGAAGTTTGTGTGTACATGTGCCCAGGCTGCAAACCACTATGAGAGACCCCACTGATCCCTGCTGTCCTGTGGAGGAGGAGCTCCAGGAATGCAGAGTGCTAGGGCCTCAGT**T-3’**

**FSHB 3’-UTR anti-sense sequence (partial):**

**5’-CTAGA**ACTGAGGCCCTAGCACTCTGCATTCCTGGAGCTCCTCCTCCACAGGACAGCAGGGATCAGTGGGGTCTCTCATAGTGGTTTGCAGCCTGGGCACATGTACACACAAACTTCTTGGACGTCTTGGTCCTTCAGACAAGGGTAGGAAGCATGAAATGTCCACTGCTCT**C-3’**

**GNAI3 3’-UTR sense sequence (partial):**

**5’-TCGAG**AGAGGGTGGATGTTAATAAAAGTTATTACGGTGTGGAGTTTCGAGACCAGACTTCTTTTGCTGTCTCATTGGGCAGTTGCAAGCATGAACGGGACCAGGGAATGGCAGCGGCATGCAGAATCTTAGCACTCTTTAGCACAATCTTTTGTATTAAGGAACT**T-3’**

**GNAI3 3’-UTR anti-sense sequence(partial):**

**5’-CTAGA**AGTTCCTTAATACAAAAGATTGTGCTAAAGAGTGCTAAGATTCTGCATGCCGCTGCCATTCCCTGGTCCCGTTCATGCTTGCAACTGCCCAATGAGACAGCAAAAGAAGTCTGGTCTCGAAACTCCACACCGTAATAACTTTTATTAACATCCACCCTCT**C-3’**
